# Family resilience and its influencing factors among advanced cancer patients and their family caregivers: a multilevel modeling analysis

**DOI:** 10.1186/s12885-023-11101-z

**Published:** 2023-07-04

**Authors:** Panpan Cui, Jiaoxia Shi, Shifeng Li, Mikiyas Amare Getu, Ruibo Wang, Changying Chen

**Affiliations:** 1 Nursing department, Henan Provincial People’s Hospital, People’s Hospital of Zhengzhou University, Zhengzhou, China; 2 School of Nursing, Zhengzhou University, Zhengzhou, China; 3 Medical Oncology, Jiaozuo People’s Hospital, Jiaozuo, China; 4 Medical Oncology, Xinyang Central Hospital, Xinyang, China; 5 School of Nursing, Woldia University, Weldiya, Ethiopia; 6 The First Affiliated Hospital of Zhengzhou University, No. 1 Jianshe Dong Road, Zhengzhou, China; 7 Institute for Hospital Management of Henan Province, Zhengzhou, China

**Keywords:** Advanced cancer, Caregivers, Influencing factors, Dyads, Family resilience

## Abstract

**Background:**

Cancer is highly prevalent worldwide. Family resilience is a positive variable that helps families burdened by advanced cancer to cope effectively. This study aimed to describe the family resilience of advanced cancer patients and caregivers in dyads and identify its influencing factors at the individual and dyadic levels.

**Methods:**

This multisite cross-sectional study was conducted in oncology units in five tertiary hospitals in China. A total of 270 advanced cancer patient-caregiver dyads were recruited between June 2020 and March 2021. Patients’ and caregivers’ family resilience was measured by the Family Resilience Assessment Scale. Data on potential influencing factors, including demographic and disease-related characteristics as well as family sense of coherence, psychological resilience, perceived social support, symptom burden, and caregiver burden, were collected. Multilevel modeling analysis was adopted to control for the interdependence of the dyads.

**Results:**

A total of 241 dyads were included in the data analysis. The mean ages of patients and caregivers were 53.96 (SD 15.37) and 45.18 (SD 13.79) years, respectively. Most caregivers were spouses and adult children (45.6% and 39.0%, respectively). Patients reported a higher mean family resilience score than caregivers (152.56 vs. 149.87, respectively). Undergoing fewer than two types of treatment and a lower symptom burden of patients predicted higher patient (*B* = -9.702, -0.134, respectively) and caregiver (*B* = -5.462, -0.096, respectively) family resilience. Patients also reported higher family resilience under the following conditions: 1) were on a medical insurance plan other than the new rural cooperative medical system (*B* = 6.089), 2) had a better family sense of coherence (*B* = 0.415), 3) whose caregivers were unmarried (*B* = 8.618), perceived lower social support (*B* = -0.145) and higher psychological resilience (*B* = 0.313). Caregivers who were ≤ 44 years old (*B* = -3.221), had similar previous caregiving experience (*B* = 7.706), and had a stronger family sense of coherence (*B* = 0.391) reported higher family resilience.

**Conclusions:**

Our findings highlight the importance of adopting a dyadic approach when caring for advanced cancer patients and their caregivers. Dyadic longitudinal research is suggested to discover more modifiable factors of family resilience and tailored interventions are needed to obtain optimal dyadic outcomes.

**Supplementary Information:**

The online version contains supplementary material available at 10.1186/s12885-023-11101-z.

## Introduction

Cancer morbidity and mortality are trending upward worldwide and in China [1]. The estimated deaths caused by cancer were almost 10 million globally and 3 million in China in 2020 [1], meaning millions of cancer patients reach an advanced disease stage each year. Advanced cancer patients refer to those who are diagnosed with stage III ~ IV cancer and have no response to the curative anticancer treatment, combined with a variety of symptoms and gradually deteriorating conditions [2]. Among Chinese patients with known stage at diagnosis, 52.8% are diagnosed with advanced stage cancer [3]. Family members often become caregivers from the onset of cancer diagnosis and provide support for their loved ones, which can be a heavy burden. Advanced cancer families face the dual challenges of the high symptom burden of patients [4] and the high caregiving burden of caregivers [5], resulting in family vulnerability [6, 7].

To enhance the family’s coping ability and reduce vulnerability, family resilience may play an important role [8]. Family resilience, first proposed by McCubbin [9] and developed by Walsh [10], focuses on how families identify strengths in adversity and bounce forward. Walsh’s Family Resilience Framework includes three key processes: the family belief system, family organization process, and family communication process [11]. Based on this framework [11], strengthening families’ proactive attitude toward problems and fully mobilizing their problem-solving ability have the potential to help them effectively cope with cancer-related stress. The fact that different family members have different perceptions of family characteristics highlights the importance of understanding family resilience from the perspective of patients and caregivers at the dyadic level.

Previous studies have shown that family resilience can positively predict the quality of life of cancer patients [12] and principal caregivers [13]. In addition, family resilience may contribute to less fear of cancer recurrence [14] and negative emotion [15, 16] while improving patients’ family communication and caregiver positivity [17]. Despite these benefits, cancer patients and caregivers may experience low to moderate levels of family resilience [18, 19]. The vast majority of the participants in previous studies were patients with stage I~III cancer: some studies included only a small proportion of patients with stage IV cancer or none at all. The status quo of family resilience for advanced cancer dyads, especially stage IV cancer dyads, needs further investigation.

Several factors were disclosed to have an impact on family resilience in the context of cancer, including demographic characteristics (e.g., age, income, education, place of residence) [20–22], patients’ clinical characteristics (e.g., type of tumor, duration, and severity of disease, treatment type) [20, 22–24] and other factors (i.e., social support, psychological resilience) [25, 26]. However, previous studies have mainly revealed the determinants of family resilience solely at the patient [20, 22] or caregiver level [21–23]. Few studies have included patients and caregivers concurrently and described family resilience at the dyadic level. Additionally, studies including both patients and caregivers have exclusively examined the family resilience of the individual population [27]. Moreover, family sense of coherence, which originated from the individual sense of coherence proposed by Antonovsky [28], reflects the family perspective and appraisal of stressful situations. According to prior studies [29–31], it is a potential determinant of family well-being but its relationship with family resilience has not yet been examined. Previous qualitative studies indicated that family stress (i.e., patients’ symptom burden, caregiver burden) may be a potential influencing factor of family resilience, which has not been fully examined by quantitative research [32]. This study aimed to fill these gaps. Perceived family resilience for both patients and caregivers and influencing factors were examined in our study.

The specific aims of this study were to (i) assess the level of family resilience of advanced cancer patients and their family caregivers in dyads and (ii) identify factors influencing family resilience at the individual and dyadic levels. We hypothesized that patients’ and caregivers’ perceived family resilience interacted with each other and were influenced by individual-level and dyadic-level factors.

### Theoretical basis

**Fig. 1:**
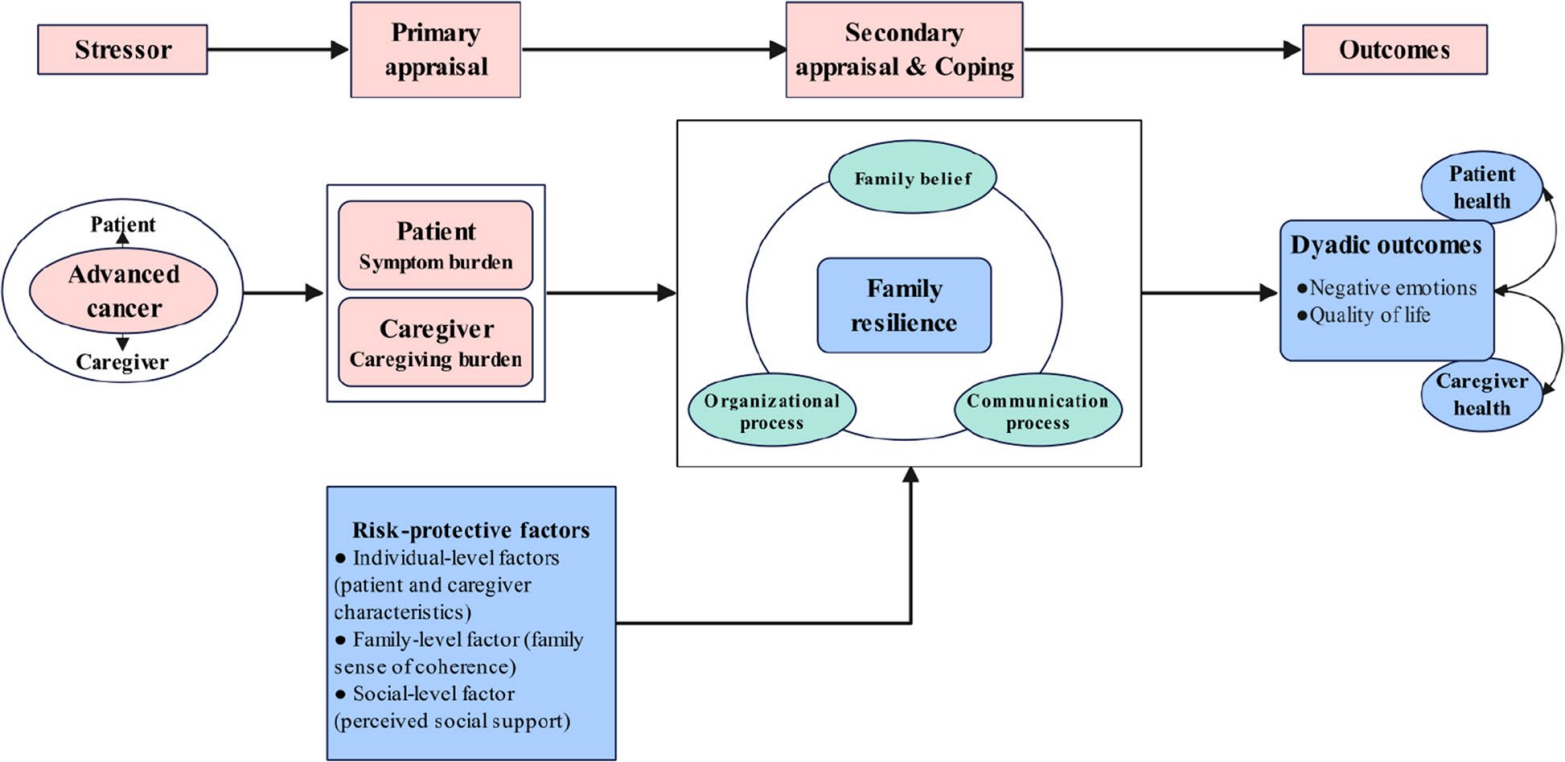
The theoretical framework

The theoretical framework was built based on several theories. The first is Lazarus' cognitive appraisal theory of stress, which emphasizes the effect of cognitive appraisal and coping on individual outcomes [33]. According to the theory, primary appraisal means what is at stake in an encounter with cancer, such as patients’ symptom burden and caregivers’ burden, while secondary appraisal depends on coping options. Secondary appraisal and coping coincides with the connotation of family resilience [34]. The second is the theory of dyadic illness management by Lyons [35], which stressed the risk and protective factors that affect dyadic appraisal and dyadic management behaviors and then improve dyadic outcomes. The third is Walsh’s Family Resilience Framework, which consists of three key processes in family coping. The theoretical framework of this study is shown in Fig. 1.

## Materials and methods

### Design and settings

This was a multisite cross-sectional study conducted between June 2020 and March 2021 in oncology units in five tertiary hospitals in Henan Province, China.

### Participants

Advanced cancer patients and their family caregivers were included in the study via a convenience sampling method. The inclusion criteria for patients were as follows: (1) older than 18; (2) a stage IV cancer, including common malignant solid tumors (i.e., lung cancer, breast cancer, esophageal cancer, colorectal cancer) and malignant blood tumors (i.e., lymphoma); (3) an education level of primary school or more; and (4) provided informed consent. Patients were excluded if they (1) had other serious physical illnesses or unstable physical conditions or (2) had serious mental illness or cognitive impairment. Family caregivers were enrolled if they (1) were at least 18 years old; (2) were identified by the patients as the main informal unpaid caregiver; (3) provided continuous care and support for patients at home and during hospitalization and participated in medical decisions for patients; (4) had an education level of primary school or more; and (5) provided informed consent. Paid caregivers and those who had severe mental illness or cognitive impairment were excluded.

For general multilevel models, 30, 50, 100, and 200 have been suggested in previous sample size guidelines; regarding dyad studies, the suggested sample size is more than 50 dyads to obtain reliable and valid estimates when there are no singletons [36]. The sample size was set at 200 in the current investigation, yielding a required sample of 250 dyads considering 20% invalid questionnaires.

### Data collection procedures

Data were collected with a self-administered questionnaire completed by advanced cancer patients and their caregivers, who were enrolled and given unified instructions by five trained research assistants (one in each hospital) to reduce potential investigator bias. All research assistants were registered nurses. The participants completed the questionnaire after informed consent was obtained. The patient and the caregiver completed their questionnaires separately. For those who could not complete the questionnaire by themselves, the research assistants helped by reading the items and recording the answers objectively. The clinical characteristics of patients were extracted from the medical record system by research assistants. Only complete dyads were included in the data analysis; if one member of the dyad failed to participate, the other member was not included, either. To improve engagement and ensure the quality of the participants’ responses, we distributed the questionnaires in the afternoon when there less treatment was conducted. In addition, research assistants were present and ready to explain any confusing items to ensure that the participants fully understood the items before completing the questionnaire. The assistants checked the questionnaires for missing items and asked participants to complete any omissions they found. Questionnaires with more than 10% missing items or those with patterned responses were discarded.

### Measurement

#### Sociodemographic and clinical characteristics

The questionnaire investigated the demographic characteristics of advanced cancer patients and their family caregivers, including age, sex, working status, marital status, and monthly household income per capita. Patients reported their place of residence, living conditions, level of understanding about their disease, and perception of disease severity.

Chart reviews were conducted for clinical characteristics of patients, including payment type for medical expenses, primary cancer, time since advanced cancer diagnosis, type of treatments, comorbidities, using the Charlson comorbidity index (CCI) and the Eastern Cooperative Oncology Group performance status (ECOG PS). Patients were categorized according to their ECOG PS, which consists of five grades from 0 “activity ability completely normal” to 4 “bedridden and unable to take care of oneself” [37]. An ECOG PS threshold of 2 is widely used, and 0–2 represents good performance status [38]. Caregivers were asked to report their relationship with patients, the presence of chronic conditions, whether they had similar caregiving experience, type (care for patients alone or with secondary caregivers) and length of caregiving, and caregiving hours per day.

#### Family resilience

Family resilience was measured in advanced cancer patients and caregivers by Sixbey’s Family Resilience Assessment Scale (FRAS) [39]. It is a 54-item questionnaire with six dimensions: family communication and problem-solving (FCPS, 27 items), utilization of social and economic resources (USER, 8 items), maintenance of a positive outlook (MPO, 6 items), family connectedness (FC, 6 items), ability to make meaning of adversity (AMMA, 3 items) and family spirituality (FS, 4 items). Participants responded on a 4-point Likert scale, 1 for strongly disagree to 4 for strongly agree; thus, the total score of the scale ranged from 54 to 216. Higher scores indicate higher levels of family resilience. The simplified Chinese version of the FRAS contained 51 items after cultural adaptation, and the Scale-level Content Validity Index (S-CVI) was 0.97, while the Cronbach’s α and test–retest reliability among cancer families were 0.944 and 0.917, respectively [40]. The C-FRAS had a Cronbach’s α of 0.941 for patients and 0.929 for caregivers in the current study.

#### Family sense of coherence

The family sense of coherence was measured in advanced cancer patients and caregivers by the short form of the Family Sense of Coherence Scale (FSOC-S), which has 12 items and one dimension [41]. The items are scored on a 7-point Likert scale from 1 to 7. The total score ranged from 12 ~ 84, with higher scores indicating a better family sense of coherence. The Cronbach’s α of the Chinese version of the FSOC-S was 0.83, and the test–retest reliability was 0.75, while all items had a CVI of more than 0.9 [42]. The FSOC-S had Cronbach’s α values of 0.827 and 0.760 for patients and caregivers, respectively.

#### Psychological resilience

Psychological resilience was measured in family caregivers by the 10-item Connor-Davidson Resilience Scale (CD-RISC-10). It was developed by Campbell-Sills and has 10 items with one dimension [43]. Items are rated from 0 “not true at all” to 4 “true nearly all the time”. The total scores ranged from 0 ~ 40, with higher scores suggesting better individual resilience. The Cronbach’s α and test–retest reliability of the Chinese version among cancer caregivers was 0.877 and 0.73, respectively, while the CVI for all items scored between 0.83 and 1 [44]. The Cronbach’s α in our sample was 0.906.

#### Perceived social support

The Chinese version of the 12-item Perceived Social Support Scale (PSSS), based on the original scale developed by Zimet [45], was administered to family caregivers to measure social support. The Chinese version of the PSSS has two factors, indicating two sources of social support: internal family (4 items) and external family (8 items) [46]. Participants responded on a 7-point Likert scale, from 1 for very strongly disagree to 7 for very strongly agree. The total scores ranged from 12 ~ 84, with higher scores indicating more perceived social support. The Cronbach’s α of the PSSS was 0.949 in our sample.

#### Symptom burden

The Edmonton Symptom Assessment System (ESAS) was selected to measure the symptom burden in advanced cancer patients [47]. It consists of nine common symptoms and the option of a tenth symptom. All items used an 11-point numerical rating method and ranged from 0 for symptom absent or best condition to 10 for worst possible condition. The Chinese version of the ESAS demonstrated acceptable internal consistency (Cronbach’s α = 0.72) and good concurrent validity (correlation coefficients between ESAS symptom scores and M.D. Anderson Symptom Inventory scores ranged from 0.70 to 0.96) [48]. The Cronbach’s α was 0.909 in the current study.

#### Caregiver burden

The 22-item Zarit Burden Interview (ZBI) was used to measure caregiver burden [49]. It comprises two factors, namely, personal burden and responsibility burden. All items are assigned from 0 (almost none) to 4 (always) points, with a total score of 0 ~ 88. Higher scores indicate a worse burden. The Chinese version of the ZBI showed good construct validity and internal consistency (Cronbach’s α = 0.87) [50]. ZBI had a Cronbach’s α of 0.914 in the current study.

### Statistical analysis

Epidata v 3.1 was used for data collation by two researchers (Cui P, Wang R) to ensure accuracy. Descriptive analyses were conducted for the characteristics of the respondents. All variables were treated as categorical variables. Cutoff points for age and time since advanced cancer diagnosis were determined by the median value to ensure balance in each stratum. Independent *t* tests and one-way ANOVA were adopted to examine the differences in the family resilience of patients and caregivers corresponding to different characteristics at the individual and dyad levels. Based on the distribution of data, Pearson or Spearman correlation analyses were used to explore the relationships between continuous independent variables (e.g., symptom burden, caregiver burden) and family resilience at the dyad level. All of the analyses were performed with IBM SPSS Version 21.0 (IBM Corporation, Armonk, NY).

Multilevel modeling was adopted to explore the factors influencing family resilience at the individual and dyadic levels to control for interdependence in the data and to identify actor effects (e.g., characteristics of advanced cancer patients associated with patients’ family resilience) and partner effects (e.g., characteristics of advanced cancer patients associated with caregivers’ family resilience) [51–53].

First, the level-1 unconditional (within-dyad) model was run, which represented family resilience for both advanced cancer patients and their caregivers as the sum of a latent score plus a residual term that seized measurement error. The measurement error represented within-dyad random effects (i.e., variability in the average levels of patients’ and caregivers’ family resilience). In the level-1 model, the advanced cancer patient-caregiver dyad is the unit of analysis rather than the individual cancer patient or caregiver. The level-1 model also provides a tau correlation, which represents the interdependence of family resilience within dyads.

A chi-square test was then performed to determine whether there was statistically significant variability around the average family resilience scores across dyads. If there was, independent variables identified from a priori testing (statistically significant in the correlation analyses and univariate analyses) were added to obtain an adjusted model, that is, a level-2 (between-dyad) model, to explain this variability. In the adjusted level-2 model, latent family resilience scores in the level-1 model served as dependent variables, and two simultaneous regression equations were run for patients and caregivers to examine the role of independent variables. Continuous independent variables were mean-centered. Actor and partner effects were examined, which were described by unstandardized regression coefficients (B) and their standard errors (SE). The α values were two-tailed and the *P* value was set at 0.05 for statistical significance. Hierarchical Linear and Nonlinear Modeling (HLM) v8 (Scientific Software International, Inc.) was used to perform the analysis with full information maximum likelihood estimation.

## Results

### Sociodemographic and clinical characteristics

**Fig. 2:**
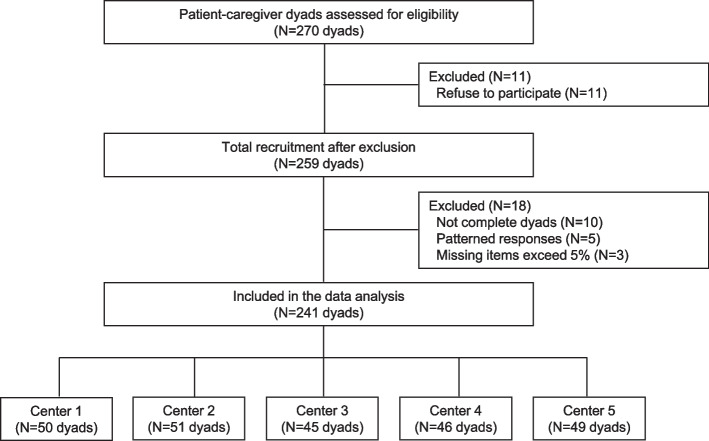
Flow diagram of participants

A total of 270 dyads were recruited (54 dyads from each center), and the questionnaires of 241 dyads remained in the final analyses, for an 89.3% response rate. The participant selection process is shown in Fig. 2.

The characteristics of the patients and caregivers are shown in Tables 1 and 2. The median ages of the patients and caregivers were 56 and 44 years, respectively. The sex proportions for patients and caregivers were different, as the proportion of males was slightly higher for patients and the proportion of females was higher for caregivers. Both patients and caregivers were predominantly married (85.5% and 87.1%, respectively) but not necessarily to one another, as 39.0% of caregivers were adult children. Patients were less educated than caregivers. Solid tumors (72.6%) were the predominant type of primary cancer, including lung cancer (51/241, 21.2%), breast cancer (22/241, 9.1%), and digestive system cancers (66/241, 27.4%); blood tumors accounted for 27.4% (66/241), comprising mainly lymphoma (55/241, 22.8%).

**Table 1:** Sample characteristics (*N* = 241dyads)

**Variables**	**Patient/n (%)**	**Caregiver/n (%)**
**Age (years)**		
M ± SD	53.96 ± 15.37	45.18 ± 13.79
Median	56	44
**Sex**		
Male	126 (52.3)	111 (46.1)
**Marital status**		
Married	206 (85.5)	210 (87.1)
Single/Divorced/Widowed	35 (14.5)	31 (12.9)
**Education**		
≤ Middle school	138 (57.3)	107 (44.4)
> Middle school	103 (42.7)	134 (55.6)
**Employment status**		
Employed	43 (17.8)	106 (44.0)
Unemployed/retired	198 (82.2)	135 (56.0)
**Place of residence**		
Rural	115 (47.7)	-
Urban	126 (52.3)	-
**Relationship patient-caregiver**		
Spouse	-	110 (45.6)
Nonspouse	-	131 (54.4)
Adult children	-	94 (39.0)
Others	-	37 (15.4)
**Living with spouse**		
Yes	180 (74.7)	-
No	61 (25.3)	-
**Average household income per capita**		
≤ 3000	133 (55.2)	124 (51.4)
> 3000	108 (44.8)	117 (48.6)
**Payment type for medical expenses**		
NCMS	106 (44.0)	-
URBMI	46 (19.1)	-
UEBMI	84 (34.8)	-
At own expenses	5 (2.1)	-
**Caregiver with a chronic condition**		
Yes	-	45 (18.7)
No	-	196 (81.3)
**Similar previous caregiving experience**		
Yes	-	61 (25.3)
No	-	180 (74.7)
**Care for the patient alone**		
Yes	-	140 (58.1)
No	-	101 (41.9)
**Length of care**		
< 6 months	-	120 (49.8)
6 ~ 12 months	-	64 (26.5)
> 12 months	-	57 (23.7)
**Caregiving hours per day**		
< 6 h	-	80 (33.2)
6 ~ 12 h	-	70 (29.0)
12 ~ 18 h	-	31 (12.9)
18 ~ 24 h	-	60 (24.9)
**Primary cancer**		
Solid tumor	175 (72.6)	-
Hematologic tumor	66 (27.4)	-
**Time since advanced cancer diagnosis/month**		
≤ 8 months^┾^	126 (52.3)	-
> 8 months	115 (47.7)	-
**Types of treatment**		
≤ 2 types	211 (87.6)	-
> 2 types	30 (12.4)	-
**Understanding level of the disease**		
Not at all	12 (5.0)	-
A little	119 (49.4)	-
Some	63 (26.1)	-
Very much	47 (19.5)	-
**Perception of disease severity**		
Not at all	7 (2.9)	-
A little	94 (39.0)	-
Some	93 (38.6)	-
Very much	47 (19.5)	-
**Comorbidities**		
Yes	56 (23.2)	-
No	185 (76.8)	-
**ECOG PS**		
≤ 2	201 (83.4)	-
> 2	40 (16.6)	-

*M* Mean, *SD* Standard deviation, *NCMS* New rural cooperative medical system, *ECOG PS* Eastern Cooperative Oncology Group performance status, *URBMI* Urban residents' basic medical insurance system, *UEBIM* Urban employees' basic medical insurance system. ^┾^: The median time since advanced cancer diagnosis was 8 months

**Table 2:** Patient and caregiver scores on various scales (*N* = 241 dyads)

Variables	M ± SD		*t*	*P*
	**Patient**	**Caregiver**		
**Family resilience (FRAS)**	152.56 ± 15.11	149.87 ± 13.97	2.546	**0.012**
FCPS	76.35 ± 8.80	74.33 ± 7.99	3.245	**0.001**
USER	23.58 ± 3.25	23.21 ± 2.91	1.706	0.089
MPO	18.61 ± 2.58	28.34 ± 2.25	1.573	0.117
FC	16.14 ± 1.99	16.00 ± 1.93	1.002	0.317
FS	8.39 ± 2.67	8.76 ± 2.73	-1.962	0.051
AMMA	9.50 ± 1.28	9.24 ± 1.40	2.500	**0.013**
**Family sense of coherence (FSOC-S)**	61.12 ± 11.31	59.40 ± 11.01	2.242	**0.026**
**Symptom burden (ESAS)**	24.53 ± 18.65	-		
**Caregiver burden (ZBI)**	-	30.31 ± 13.50		
**Resilience (CD-RISC-10)**	-	31.22 ± 9.31		
**Social support (PSSS)**	-	58.43 ± 13.15		

*M* Mean, *SD* Standard deviation, *FRAS* Family resilience assessment scale, *FCPS* Family communication and problem solving, *USER* Utilization of social and economic resources, *MPO* Maintaining a positive outlook, *FC* Family connectedness, *FS* Family spirituality, *AMMA* Ability to make meaning of adversity, *FSOC-S* Family sense of coherence scale-short form, *ESAS* Edmonton Symptom Assessment System, *ZBI* Zarit Burden Interview, *CD-RISC*-10 10-item Connor-Davidson Resilience Scale, *PSSS* Perceived Social Support Scale

### Bivariate analysis of different variables at the individual and dyadic levels

At the individual level, patients’ family resilience was positively correlated with their family sense of coherence (*r* = 0.345) but negatively correlated with their symptom burden (*r* = -0.261). Caregivers’ family resilience had a positive correlation with their family sense of coherence and perceived social support (*r* = 0.345 and 0.289, respectively) but had a negative correlation with their caregiver burden (*r* = -0.248).

At the dyad level, there was a positive correlation between patients’ and caregivers’ family resilience (*r* = 0.476). Patients’ family sense of coherence was positively correlated with caregivers’ family resilience (*r* = 0.163) while caregivers’ family sense of coherence and psychological resilience were positively correlated with patients’ family resilience (*r* = 0.136 and 0.138, respectively). In addition, patients’ symptom burden was negatively associated with caregivers’ family resilience (*r* = -0.175), as shown in Table 3.

**Table 3:** Bivariate analysis of patients' and caregivers' different variables (*N* = 241 dyads)

Variables	Patient			Caregiver				
	**FRAS**	**FSOC-S**	**ESAS**	**FRAS**	**FSOC-S**	**ZBI**	**CD-RISC-10**	**PSSS**
**FRAS** (patient)	1							
**FSOC-S** (patient)	0.345**	1						
**ESAS** (patient)	-0.261**	-0.291**	1					
**FRAS** (caregiver)	0.476**	0.163*	-0.175**	1				
**FSOC-S** (caregiver)	0.136*	0.409**	-0.102	0.345**	1			
**ZBI** (caregiver)	-0.076	-0.180**	0.117	-0.248**	-0.298**	1		
**CD-RISC-10** (caregiver)	0.138**	0.165*	0.014	0.106	0.394**	-0.022	1	
**PSSS** (caregiver)	0.072	0.221**	-0.034	0.289**	0.515**	-0.222**	0.293**	1

*FRAS* Family Resilience Assessment Scale, *FSOC-S* Family Sense of Coherence-short form, *ESAS* Edmonton Symptom Assessment System, *ZBI* Zarit Caregiver Burden Interview, *CD-RISC*-10 10-item Connor-Davidson Resilience Scale, *PSSS* Perceived Social Support Scale

^*^ *P* < 0.05; ** *P* < 0.01

### Univariate analyses of individual and dyadic factors associated with family resilience

At the individual level, patients who were employed, had a higher monthly household income per capita, paid for medical expenses with medical insurance other than NCMS, were diagnosed with advanced cancer for less than 8 months, underwent less than two types of treatment, and had a higher level of understanding about their disease reported higher family resilience scores (*P* < 0.05). Caregivers who were less than 44 years old and had similar previous caregiving experience had higher family resilience scores (*P* < 0.05).

At the dyad level, patients were likely to have lower levels of family resilience when their caregivers were married, reported a monthly household income per capita of less than 3000 RMB, and had a length of care of more than 6 months (*P* < 0.05). Caregivers reported higher levels of family resilience when patients underwent fewer than two types of treatment (*P* < 0.01). See Additional file 1.

### Multilevel models predicting family resilience

In the unconditional level-1 model, patients and caregivers reported a moderate level of family resilience (152.56 vs. 149.87, respectively). Statistically significant variability (*P* < 0.001) around the average family resilience scores for both the patients and the caregivers suggested notable heterogeneity in family resilience across the dyads. The tau correlation between patient and caregiver family resilience was 0.80, indicating high interdependence of family resilience within dyads. See Table 4.

**Table 4:** Unconditional multilevel model fixed and random results for patient and caregiver family resilience (*N* = 241 dyads)

	*B*	*SE*	*P*
**Fixed effects**			
Patient intercept	152.56	0.97	< 0.001
Caregiver intercept	149.87	0.90	< 0.001
**Random effects**	Variance Components	χ^2^	*P*
Patient	114.86	482.52	< 0.001
Caregiver	81.43	412.12	< 0.001

*B* Unstandardized coefficient, *SE* Standard error

Influencing factors were identified at the individual and dyad levels in the adjusted model, as shown in Table 5. Patients’ symptom burden and types of treatment had both actor effects and partner effects in predicting patients’ and caregivers’ family resilience. Patients’ and caregivers’ family resilience scores were lower when patients reported higher symptom burden (*B* = -0.134, -0.096, respectively) and underwent more than two types of treatment (*B* = -9.702, -5.462, respectively). Both patients’ and caregivers’ family sense of coherence had statistically significant actor effects on their own family resilience (*B* = 0.415 and 0.391, respectively). Patients who paid medical expenses with NCMS reported a lower level of family resilience (*B* = 6.089). Caregivers’ marital status, individual resilience, and perceived social support had partner effects on patients’ family resilience, with unmarried (*B* = 8.618) and higher individual resilience (*B* = 0.313) indicating higher levels of patients’ family resilience. Surprisingly, patients reported a lower level of family resilience when their caregivers had more social support (*B* = -0.145). Caregivers older than 44 were at risk of reporting a lower level of family resilience (*B* = -3.221), while those with similar previous caregiving experience reported higher levels of family resilience (*B* = 7.706).

**Table 5:** Adjusted multilevel model predicting for patient and caregiver family resilience (fixed and random effects) (*N* = 241 dyads)

	**FRAS (Patient)**			**FRAS (Caregiver)**		
	***B***	***SE***	***P***	***B***	***SE***	***P***
**Fixed effects**						
***Intercept***	125.85	6.97	< 0.001	140.76	7.23	< 0.001
***Patient variables***						
Employment status (employed ^a^)	-0.253	2.336	0.914	1.101	2.300	0.633
Monthly household income per capita (≤ 3000 RMB ^a^)	0.650	1.889	0.731	-1.967	1.981	0.322
Payment type for medical expenses (NCMS ^a^)	6.089	1.720	** < 0.001**	2.629	1.753	0.135
Time since advanced cancer diagnosis (≤ 8 months ^a^)	-2.791	1.779	0.118	0.878	1.726	0.611
Understanding level of the disease	1.016	1.055	0.337	0.262	1.002	0.794
Types of treatment (≤ 2 types ^a^)	-9.702	2.264	** < 0.001**	-5.462	2.311	**0.019**
Symptom burden (ESAS)	-0.134	0.042	**0.002**	-0.096	0.040	**0.016**
Family sense of coherence (FSOC-S)	0.415	0.094	** < 0.001**	-0.040	0.089	0.651
***Caregiver variables***						
Age (≤ 44 years old ^a^)	1.082	1.715	0.529	-3.221	1.578	**0.042**
Marital status (married ^a^)	8.618	2.879	**0.003**	-0.198	2.871	0.945
Similar previous caregiving experience (no ^a^)	2.176	1.944	0.264	7.706	1.854	** < 0.001**
Length of care	-0.721	1.116	0.519	-0.141	1.015	0.890
Monthly household income per capita (≤ 3000 RMB ^a^)	2.461	1.920	0.201	0.817	1.926	0.672
Family sense of coherence (FSOC-S)	-0.034	0.087	0.696	0.391	0.104	** < 0.001**
Psychological resilience (CD-RISC-10)	0.313	0.092	** < 0.001**	0.042	0.107	0.694
Caregiver burden (ZBI)	-0.058	0.064	0.364	-0.090	0.074	0.220
Perceived social support (PSSS)	-0.145	0.073	**0.048**	0.100	0.090	0.267
**Random effects**	Variance Components	χ^2^	*P*			
Patient	65.61	386.22	< 0.001			
Caregiver	65.18	385.19	< 0.001			

^a^Reference group. *B* Unstandardized coefficient, *SE* Standard error, *FRAS* Family resilience assessment scale, *FSOC*-*S* Family sense of coherence scale-short form, *ESAS* Edmonton Symptom Assessment System, *ZBI* Zarit Burden Interview, *CD-RISC*-10 10-item Connor-Davidson Resilience Scale, *PSSS* Perceived Social Support Scale, *NCMS* New rural cooperative medical system. Coefficients are adjusted for the influence of all multivariate model factors. Additional file 2 shows the assignment of the independent variables and the reference categories

## Discussion

The current study describes family resilience and examines the influencing factors while controlling for interdependence among advanced cancer patient-caregiver dyads. Our findings highlight the importance of dyadic perspectives when assessing family resilience and related factors in the context of advanced cancer because we found that the characteristics of a member of the dyad could influence the perceived family resilience of the other. Paying attention to these factors may facilitate the detection of families at risk of vulnerability and provide targeted support.

Our study demonstrated that patients’ and caregivers’ family resilience was at a moderate level, similar to that of Chen’s study [19] but lower than that of Li’s study [27]. It may be that patients in previous studies were diagnosed with stage I~III cancer, while our study only included cancer patients in stage IV who might be more dependent on caregivers. The higher level of family resilience perceived by patients may be related to more caregiver support. However, caregivers providing more support may perceive a greater caregiving burden and thus a relatively low level of family resilience [54].

### Influencing factors predicting patients’ family resilience

We identified several patient characteristics associated with patient family resilience (actor effects) and with caregiver family resilience (partner effects), as well as caregiver characteristics associated with their family resilience (actor effects) and patient family resilience (partner effects).

#### Patients’ payment type for medical expenses (actor effect)

We found that patients perceived lower levels of family resilience when they paid medical expenses with NCMS, which was not demonstrated in previous studies. It may be that most patients with NCMS live in rural areas, and the NCMS designated hospitals are mainly secondary or lower level health care facilities. The complexity of advanced cancer might increase the possibility of patients seeking higher level medical services in tertiary hospitals in nondesignated cities, resulting in a lower reimbursement ratio and increased out-of-pocket expenses [55]. Thus, patients may perceive a greater financial burden and the vulnerability of family coping. Evidence suggests that cancer patients lack medical insurance literacy and providing them with education about insurance coverage and out-of-pocket expenses is an important supportive care strategy [56].

#### Patients’ symptom burden and types of treatment (actor effects)

Our results indicated that patients and caregivers may be particularly at risk for lower levels of family resilience when patients suffered from a higher symptom burden and underwent more than two types of treatment, which is a consideration that has been less reported [20]. It may be that higher levels of symptom burden give rise to difficulties in self-management, resulting in a lower perception of family resilience. This may also explain the negative influence of multimodal treatments, as more treatments might mean managing multiple adverse reactions simultaneously, which may be challenging for patients. To identify vulnerable families, regular symptom burden assessment is suggested, and the families of patients who undergo three or more treatments require the most attention.

#### Patient-reported family sense of coherence (actor effect)

Patients reported higher family resilience when they perceived a stronger family sense of coherence, the connotations (comprehensibility, manageability, and meaningfulness) of which indicated that patients perceived the family pressure caused by cancer as understandable, regarded it as a challenge rather than a burden, had a sense of control over family changes with their family resources, and found meaning in coping with cancer [28]. In addition, these connotations are theoretically similar to the family belief system (e.g., making meaning of adversity, a positive outlook) in the Family Resilience Framework [11]. Endowing advanced cancer-related stress with meaning helps families develop new ways of thinking and better adapt to challenges [57]. Family strength-based interventions are suggested targeting family sense of coherence with key points proposed by previous theories.

#### Caregiver’s marital status (partner effect)

Our results suggested that patients may be at risk for lower levels of family resilience when their caregivers are married. As married caregivers are predominantly spouses or adult children, they may assume other family roles in addition to the caregiving task and are more prone to time disturbance and health problems [58]. Thus, time spent in caregiving may decline, and patients may perceive less family support. Additionally, in our sample compared to unmarried caregivers, married caregivers had lower education, implying a weaker ability to obtain information support and communicate with others [59]. Thus, they may provide patients with less decision-making and emotional support, and patients may perceive a relatively low level of family resilience.

#### Caregiver-reported psychological resilience/perceived social support (partner effect)

Higher psychological resilience of caregivers positively predicted patients’ family resilience. It has previously been demonstrated in a systematic review that caregivers with stronger resilience can flexibly adapt to the caregiving role and cancer-related changes in family life, evaluate stress positively, and have a strong ability to obtain knowledge of disease and caregiving skills from health care professionals, literature and networks [60], which means that caregivers are better prepared to manage stress and provide an effective support source for patients [61]. Thus, patients perceive stronger coping abilities in their family and a higher level of family resilience.

Patients reported a lower level of family resilience when their caregivers perceived more social support. This is surprising because higher levels of social support always indicate higher care ability of caregivers [62]. There might be several reasons. First, it may be that cancer is a family event, especially when it progresses to the advanced stage [63]. Most likely, caregivers take extra time to meet patients’ needs, and most family resources and acquired social support are pooled for patients [64], making caregivers of advanced cancer patients a vulnerable group. In addition, more social support may not mean higher utilization, which is conducive to good individual or family coping [65]. In this study, caregivers reported lower levels of social support than previous research targeting caregivers of stage I~III cancer patients [19] and a low score of the social resources utilization domain of family resilience. More studies are needed to understand the caregiving experience in the future to better explain the negative predictive effect of social support on patients’ family resilience. Moreover, caregivers’ perceived social support was negatively related to caregivers’ burden in this study, and the effects of social support and caregiver burden on family resilience may cancel each other out when included in the equation. Last, as social support was only reported by caregivers in the current study, future studies may include both patients’ and caregivers’ perceived social support and further explore the reason for this finding.

### Influencing factors of caregivers’ family resilience

#### Caregiver’s age (actor effect)

Younger caregiver age (≤ 44 years old) was associated with higher family resilience, which was inconsistent with prior research wherein this variable predicted lower family resilience [20]. In their study, all breast cancer patients were in the first year after cancer diagnosis and receiving active treatment [20]. Their family members might have been experiencing more disease uncertainty. In addition, younger caregivers may deal with other stressors concurrently. In this study, nearly half of the patients had been diagnosed with stage IV cancer more than eight months earlier. Caregivers might face more complicated and challenging caregiving tasks and need more informational and other support. Younger caregivers might have an advantage in information seeking [59] and thus perceive a higher level of family resilience.

#### Caregiver-reported similar caregiving experience (actor effect)

Caregivers with similar caregiving experiences reported a higher level of family resilience. It may be that caregivers obtained helpful information from their past care experience and accumulated coping strategies to deal with stressful cancer-related events [60]. In that case, uncertainty about patients’ disease trajectory and caregiving difficulties would decline, while caregiving skills would increase [66]; thus, caregivers may perceive stronger coping abilities of the family and a higher level of family resilience. Another contributor to caregivers’ higher family resilience was a better family sense of coherence. Caregivers might share with patients the same family beliefs and felt strong family connectedness in tackling advanced cancer [28, 57].

#### Patients’ symptom burden and types of treatment (partner effects)

Caregivers’ perceived family resilience was lower when patients reported a higher symptom burden and underwent more treatment. This might imply more complicated patient conditions and pose challenges for caregiving [67, 68]. Few previous studies have investigated the influence of symptom burden and types of treatment on family resilience in dyads, although one study of a sole patient population identified the impact of treatment without examining how it worked [20]. The results highlight the need to support caregivers when patients experience more symptom burden and multiple treatments.

### Clinical implications

Our findings reveal three partner effects for patients and two partner effects for caregivers after the inclusion of predicting variables for both in the multivariate model, elucidating the importance of considering the dyads as the “unit of care”, especially in the context of advanced cancer. Relying solely on individual responses may miss key factors that might highlight the risk of poor family resilience. Additionally, factors that influence the family resilience of both members of advanced cancer patient-caregiver dyads should be addressed. More attention should be given to those families bearing the characteristics found in the current study (symptom burden, treatment type, family sense of coherence, medical expenses, marital status, previous caregiving experience, psychological resilience, and perceived social support). Targeted strategies should be developed for vulnerable families.

### Strengths and limitations

Our study supplemented the evidence on the family resilience of advanced cancer patients and their caregivers at the dyadic level. By adopting proper dyadic methodologies, we obtain a more realistic estimate of the factors influencing family resilience for both patients and caregivers. However, there are several limitations. First, the cross-sectional design could not establish causal relationships and potential changes in family resilience over time could not be observed. Second, there may be response bias and recall bias. Nonresponse may be caused by higher family burden and low levels of family resilience. The lack of information about nonrespondents is a limitation. In addition, most of the variables analyzed in the study were based on self-reported data which might be susceptible to recall bias. Third, the sample was from oncology units and participants were advanced cancer patients and caregivers. The generalizability of the results may be limited to dyads with other chronic conditions. Finally, the multilevel model accounted for a moderate amount of variance in patient and caregiver family resilience but did not include other potential factors, such as dyadic coping. In addition, the coefficients of some of the influencing factors in the current study are small, such as perceived social support for patients’ family resilience; thus, interpretation of results requires caution. Future studies can adopt a longitudinal design to observe changes in family resilience at the dyadic level, and more modifiable variables could be examined to guide tailored interventions.

## Conclusions

Our study shows the reciprocal effect of patients’ and caregivers’ characteristics on their family resilience at the individual and dyadic levels. Family resilience was at a moderate level. Symptom burden, family sense of coherence, perceived social support and individual resilience were modifiable factors of better family resilience. Dyadic interventions are needed to obtain optimal outcomes for both advanced cancer patients and their caregivers.

## Supplementary Information

**Additional file 1.**

**Additional file 2.**

## Data Availability

All data generated or analyzed during this study are included in this published article and its supplementary information files. The datasets are available from the corresponding author on reasonable request.
