# Supplementary material for: Family resilience and its influencing factors among advanced cancer patients and their family caregivers: a multilevel modeling analysis
Source: BMC Cancer. 2023 Jul 4;23:623. doi: 10.1186/s12885-023-11101-z (PMC10320962; doi:10.1186/s12885-023-11101-z)
Supplement: Supplementary file 1 — Additional file 1. [file 12885_2023_11101_MOESM1_ESM.docx]

Additional file 1 Univariate analyses of the factors associated with family resilience (*N*=241 dyads)

| **Variables** | | **FRAS (M±SD)** | |  | **Variables** | | **FRAS (M±SD)** | |
| --- | --- | --- | --- | --- | --- | --- | --- | --- |
|  |  | **Patient** | **Caregiver** |  |  |  | **Patient** | **Caregiver** |
| **Patient variables** | |  |  |  | **Caregiver variables** | |  |  |
| Age | ≤56┼ | 153.80±15.39 | 149.35±15.33 |  | Age | ≤44^┽^ | 152.96±15.96 | 151.96±15.12**^*^** |
|  | >56 | 151.15±14.74 | 150.46±12.28 |  |  | >44 | 152.16±14.26 | 147.77±12.41 |
| Gender | Male | 152.93±14.82 | 149.48±13.37 |  | Gender | Male | 154.45±14.89 | 150.86±16.04 |
|  | Female | 152.16±15.49 | 150.30±14.64 |  |  | Female | 150.95±15.17 | 149.02±11.91 |
| Marital status | Married | 152.02±14.70 | 149.33±13.35 |  | Marital status | Married | 151.53±14.17***** | 149.76±12.93 |
|  | Unmarried | 155.71±17.24 | 153.03±17.04 |  |  | Unmarried | 159.55±19.23 | 150.61±19.90 |
| Education | ≤ Middle school | 152.08±13.98 | 150.20±13.40 |  | Education | ≤ Middle school | 151.20±13.74 | 148.11±13.16 |
|  | > Middle school | 153.20±16.56 | 149.44±14.75 |  |  | > Middle school | 153.65±16.10 | 151.28±14.48 |
| Employment status | Employed | 157.37±16.09***** | 151.30±16.93 |  | Employment status | Employed | 154.01±15.54 | 151.56±14.68 |
|  | Unemployed | 151.52±14.73 | 149.56±13.27 |  |  | Unemployed | 151.42±14.73 | 148.55±13.29 |
| Place of residence | Rural | 150.70±14.24 | 149.92±15.02 |  | Relationship | Spouse | 151.83±15.10 | 148.77±13.03 |
|  | Urban | 154.25±15.74 | 149.83±13.00 |  |  | Non-spouse | 153.18±15.15 | 150.79±14.70 |
| Living with spouse | Yes | 151.93±14.56 | 148.84±12.73 |  | Chronic condition | Yes | 153.31±15.03 | 149.99±14.18 |
|  | No | 154.43±16.64 | 152.90±16.86 |  |  | No | 149.29±15.22 | 149.33±13.13 |
| Average household income per capita | ≤3000 | 149.50±14.16******* | 149.80±13.37 |  | Average household income per capita | ≤3000 | 149.39±13.57****** | 148.53±13.32 |
|  | >3000 | 156.33±15.46 | 149.95±14.73 |  |  | >3000 | 155.92±15.98 | 151.29±14.54 |
| Payment type for medical expenses | NCMS | 148.27±13.39****** | 148.29±14.30 |  | Similar previous caregiving experience | Yes | 155.07±16.55 | 154.34±15.66****** |
|  | URBMI | 155.74±14.11 | 152.24±16.09 |  |  | No | 151.71±14.55 | 148.36±13.05 |
|  | UEBMI | 156.27±16.01 | 150.58±12.52 |  | Care for the patient alone | Yes | 151.46±15.35 | 148.60±14.16 |
|  | At own expenses | 151.80±23.41 | 149.60±5.51 |  |  | No | 154.08±14.73 | 151.63±13.57 |
| Primary cancer | Solid tumor | 152.99±15.46 | 149.86±14.57 |  | Length of care | <6 months | 155.01±16.24***** | 151.16±14.89 |
|  | Hematologic tumor | 151.42±24.20 | 149.91±12.32 |  |  | 6~12 months | 150.27±14.40 | 149.70±14.44 |
| Time since advanced cancer diagnosis | ≤8 months^┾^ | 154.90±15.04***** | 150.77±14.57 |  |  | >12 months | 149.98±12.63 | 147.35±10.98 |
|  | >8 months | 150.00±14.85 | 148.89±13.27 |  | Caregiving hours per day | <6h | 153.24±14.62 | 148.61±13.69 |
| Types of treatment | ≤2 types | 153.35±15.51****** | 150.48±14.57**^**^** |  |  | 6~12h | 154.13±16.37 | 151.74±14.44 |
|  | >2 types | 147.03±10.63 | 145.57±7.52 |  |  | 12~18h | 150.26±14.37 | 148.74±9.28 |
| Understanding level of the disease | Not at all | 149.58±16.45***** | 144.00±17.71 |  |  | 18~24h | 151.02±14.69 | 149.95±15.75 |
|  | A little | 152.16±13.83 | 150.96±14.53 |  |  |  |  |  |
|  | Some | 149.30±15.11 | 147.30±12.93 |  |  |  |  |  |
|  | Very much | 158.70±16.52 | 152.06±12.27 |  |  |  |  |  |
| Perception of disease severity | Not at all | 162.71±23.80 | 151.14±17.49 |  |  |  |  |  |
|  | A little | 152.12±12.02 | 151.34±12.42 |  |  |  |  |  |
|  | Some | 154.34±15.38 | 148.60±15.72 |  |  |  |  |  |
|  | Very much | 148.40±17.68 | 149.26±12.78 |  |  |  |  |  |
| Comorbidities | No | 153.43±14.36 | 150.33±13.37 |  |  |  |  |  |
|  | Yes | 149.68±17.20 | 148.36±15.82 |  |  |  |  |  |
| ECOG PS | ≤2 | 152.76±14.74 | 150.16±13.20 |  |  |  |  |  |
|  | >2 | 151.79±17.21 | 148.43±17.44 |  |  |  |  |  |
| ┼: The median age of patients was 56 years old; ┽: The median age of caregivers was 44 years old; ┾: The median time since advanced cancer diagnosis was 8 months. FRAS: Family resilience assessment scale; M: Mean; SD: Standard deviation; NCMS: New rural cooperative medical system; URBMI: Urban residents' basic medical insurance system; UEBIM: Urban employees' basic medical insurance system; ECOG PS: Eastern Cooperative Oncology Group performance status. **P*<0.05; ***P*<0.01; ****P*<0.001 | | | | | | | | |
