# Supplementary material for: Family resilience and its influencing factors among advanced cancer patients and their family caregivers: a multilevel modeling analysis
Source: BMC Cancer. 2023 Jul 4;23:623. doi: 10.1186/s12885-023-11101-z (PMC10320962; doi:10.1186/s12885-023-11101-z)
Supplement: Supplementary file 2 — Additional file 2. [file 12885_2023_11101_MOESM2_ESM.docx]

Additional file 2 Assignment of variables in the multilevel model

| Variables | Category | Value |
| --- | --- | --- |
| **Patient variables** |  |  |
| Employment status | Unemployed | 0 |
|  | Employed | 1 |
| Monthly household income per capita (RMB) | ≤3000 | 1 |
|  | >3000 | 2 |
| Payment type for medical expenses | NCMS | 0 |
|  | Others | 1 |
| Time since advanced cancer diagnosis | ≤8 months | 1 |
|  | >8 months | 2 |
| Understanding level of the disease | Not at all | 1 |
|  | A little | 2 |
|  | Some | 3 |
|  | Very much | 4 |
| Types of treatment | ≤2 types | 1 |
|  | >2 types | 2 |
| Symptom burden (ESAS) | - | Input with original value and process with value centralization |
| Family sense of coherence (FSOC-S) | - | Input with original value and process with value centralization |
| **Caregiver variables** |  |  |
| Age (years) | ≤44 | 1 |
|  | >44 | 2 |
| Marital status | Married | 0 |
|  | Unmarried | 1 |
| Monthly household income per capita (RMB) | ≤3000 | 1 |
|  | >3000 | 2 |
| Similar previous caregiving experience | No | 0 |
|  | Yes | 1 |
| Length of care | <6 months | 1 |
|  | 6~12 months | 2 |
|  | >12 months | 3 |
| Family sense of coherence (FSOC-S) | - | Input with original value and process with value centralization |
| Caregiver burden (ZBI) | - | Input with original value and process with value centralization |
| Psychological resilience (CD-RISC-10) | - | Input with original value and process with value centralization |
| Perceived social support (PSSS) | - | Input with original value and process with value centralization |
| *Note*: NCMS: New rural cooperative medical system; FSOC-S: Family sense of coherence scale-short form; ZBI: Zarit Burden Interview; CD-RISC-10: 10-item Connor-Davidson Resilience Scale; PSSS: Perceived social support scale. | | |
|  |  |  |
|  |  |  |
